# Recommendations for the early diagnosis of COPD: the AIMAR view

**DOI:** 10.1186/s40248-015-0005-4

**Published:** 2015-02-22

**Authors:** Bartolome R Celli

**Affiliations:** Division of Pulmonary, Critical Care and Sleep Medicine, Brigham and Women’s Hospital Professor of Medicine Harvard Medical School, 75 Francis Street, Boston, MA 02115 USA; Pulmonary Critical Care and Sleep Division, Brigham and Women’s Hospital, 75 Francis Street, Boston, MA 02115 USA

Chronic obstructive pulmonary disease (COPD) has the dubious distinction of being one of the few major causes of death that continues to rise in the United States and the world [1]. In that sense, its prevention, early diagnosis when clinically present, and finally its appropriate treatment should constitute a priority item in today’s health care agenda. In this sense, the recently published AIMAR’s recommendation for the early diagnosis of COPD constitutes a valuable contribution to this concerted effort [2]. Professor Nardini and colleagues are to be commended for the comprehensive effort that has made this publication possible.

Perhaps the title is a little misguided as it only describes the document as a “recommendation for diagnosis”, when in reality the content is extremely comprehensive covering not just diagnostic issues of relevance, but it also develops, in rich detail, primary and secondary prevention as well as treatment. Furthermore, it extends its recommendations to the often forgotten area of end-of-life and palliative care so important in our ageing population. Perhaps the title should have included the fact that it recommends prevention and treatment as well as diagnosis. In their modesty, the authors do not pretend this to be an evidence based document, primarily due to the fact that no evidence methodology was used, but its recommendations are very much in line with those existing in the international guidelines [3,4].

The document begins with a layout of the landscape. It attempts to relate what is known about prevalence and the burden of COPD around the world to the situation in Italy itself. Overall, it would appear that there is room to improve and in a more precise manner determine the Italian situation. It follows with a section on prevention and then with an excellent discussion on the issue of secondary screening. For this, the authors seem to favor the use of a questionnaire to select individuals likely to yield a favorable ratio of persons screened over individuals who will ultimately have the disease. The section on primary prevention not only concentrates on smoking prevention, but importantly in education and indoor and outdoor pollution. In this regard, it is important to remember that close to 20% of subjects known to have limitation to airflow in population surveys and who would qualify for a diagnosis of COPD, do not have a history of smoking or even underlying asthma [5-7]. Once again, the document emphasizes the importance of the use of spirometry in an attempt to decrease not only the problem of under-diagnosis, but the equally important and prevalent problem of misdiagnosis and miss treatment. In a series of figures and algorithmic graphs, the reader is instructed on the sequential approach to the suspected case and flow diagrams that can help orient one’s practice to achieve the best possible yield.

Perhaps one of the novelties in relation to many other documents is that the current recommendations address the potential complementary role of primary care health givers or GP’s and the specialist. It is clear to most, that given the large volume of people who smoke and who are potential candidates to develop COPD, it would be impossible to care for all patients at the specialist level. Therefore, a need exists to lay bridges that allow all caregivers to play an important role in the containment of the epidemic of COPD. Without being restrictive, this document does an excellent job in addressing this issue.

Another area that is novel, is the integration of the clinical impression to the spirometric results obtained during the forced vital capacity maneuver. Indeed, the controversy regarding whether to define COPD using a strict value of the fixed ratio or to use the predicted equations available to determine normalcy is left to the criteria of the clinician facing the patient. In this regard, I very much like the option presented, where the clinician takes into account not just the spirometric result, but incorporates his/her clinical acumen to make a definitive diagnosis. The document then addresses the grading of disease severity. This is done incorporating the current thinking that COPD is not just a disease of the lungs, but also one that affects extra-pulmonary organ systems and thus expands the possibility of using multiple domains to the well-known physiological grading that has been so useful in the past. Following the determination of the severity, the reader is led to the logical question of how is the follow up going to be implemented. In a table developed to synthesize the information, the authors present their opinion on how to achieve the goal while incorporating not only the spirometric values, but also the oximetry results, a test that has gradually become a vital sign in most offices. Whether to refer to a specialist, if the patients is being treated by a generalist, or whether the care is to remain in the hands of the primary care environment is recommended based on the severity and compromise of the individual patient.

The recommendations follow with a very good review of the elements needed to help a patient quit smoking if he or she still does and then enters into the use of the many medications now available to treat COPD. The algorithm presented as Figure number 8 is, in my opinion, very good. Not only does it provide the caregiver some leeway on the therapy to indicate and at what level, but it is also simple and direct. Most guidelines now present complex schemes that are difficult to follow and even more difficult to implement. The therapy is emphatic about the importance of considering pulmonary rehabilitation, a therapeutic tool that although accepted by everyone is not available to most patients who would benefit from the modality [8]. Long term oxygen therapy is also addressed, using a clear algorithm that has resisted the test of time and that remains the most influential treatment in terms of survival since the initial studies were completed.

A small section on home care introduces this concept, the scope of which still remains a matter of debate. Many factors play a role in the feasibility of caring for complex patients always at home. There is no question that it is the desired environment for all sick persons, but social realities and societal resources end up interplaying in the final equations of these decision. It would be fair to say that in this review, not every aspect is touched in depth (this is not the scope of the work), but at least attempts were made to cover the most important parts in a relatively brief document. In this regard, the authors need to be commended, because they end the practicum with a relatively long and important review and recommendations related to end-of-life issues and of palliative care, which as I mentioned at the beginning of this editorial, is a frequently absent discussion in most documents.

I cannot end without discussing several aspects that are absent from this document. Perhaps and most important, one that is common even to guidelines and this is the area of the co-morbidites of COPD [9]. I shall be the first to recognize that it is not easy to provide practical recommendations to all of the potential co-morbidities that may affect COPD patients, but efforts have to be made to begin to tackle this important fact. Secondly, it is not uncommon that patients with the severe disease have to face the possibility of surgery, an event that presents a risk for some, but sometimes an opportunity to improve life style or even survival itself. How to address it, and the practical approach to such events could be of use in a document such as this one. Finally, the frequent event of exacerbations is not a component of these recommendations, perhaps an area for a future supplement to this well-developed document.

My compliments to the authors and to AIMAR for having supported it. The more effort we devote to the cause, the more likely it is that we shall be successful.
